# Doctors' Fees in Germany

**Published:** 1890-03-15

**Authors:** 


### DOCTORS' FEES IN GERMANY.

For some time there has existed a considerable amount of dissatisfaction within the medical profession in Germany with regard to the old regulations for doctors' fees, which were not considered to be in accordance with the times. At a recent meeting of the doctors of Berlin and the province of Brandenburg the following resolutions were agreed to :—

1. The charges as per rate of 1815 shall be discontinued.

2. A new list of charges shall be framed for sick clubs and persons without means, with a view of getting definite regulations sanctioned by the Government.

3. With reference to persons without means, not only their actual income, but also their other means of existence, shall be considered.

4. The proposed charges are minimum charges, which can be increased where there is occasion.

5. The fees shall not be raised in cases of epidemics.

6. A court of arbitration shall be appointed, consisting of doctors and various officials, to which doubtful cases shall be referred.

The new proposal divides the above area in three classes: I. Berlin; II. Towns, villages, etc., with more than 10,000 inhabitants; and III. Towns, etc., etc., with less than 10,000 inhabitants. The charges agreed upon are as follows :—

| | I. Mks. | II. Mks. | III. Mks. |
|---|---|---|---|
| For the first visit in the doctor's residence | 3 | 2.50 | 1.75 |
| ,, subsequent visits ... ... ... | 2 | 1.50 | 1 |
| ,, every patient in the same house after the first ... ... ... ... | 1.75 | 1 | 0.75 |
| ,, visits at fixed hours ... ... ... | 5 | 4 | 3 |
| ,, night visits (between eight p.m. and seven a.m.) ... ... ... ... | 9 | 7.50 | 5 |
| ,, several doctors called in—for each ... | 10 | 8 | 5 |
| ,, letter to other doctor ... ... ... | 3 | 2.50 | 1 |
| ,, ordinary confinement ... ... ... | 7 | 5 | 3 |
| ,, accouchement, when instruments used | 15 | 10 | 7 |
| ,, an ordinary certificate ... ... ... | 3 | | |
| ,, a certificate based upon scientific examination ... ... ... ... | 10 | | |
| ,, electric séance ... ... ... ... | 2 | | |
| ,, tooth drawn ... ... ... ... | 2 | 1.50 | 1 |
| ,, obduction ... ... ... ... | 10 | 5 | 5 |
| ,, restoration to life—attempts ... ... | 15 | 10 | 8 |

Expenses for carriage refunded when distance over two kilometres (about one mile); loss of time according to the ordinary regulations for public officials. These charges only apply to sick clubs and poor persons. With regard to better positioned patients the charge becomes a matter of arrangement, or rests with the doctor.

